# The Maltine Company Prize Essay Awards

**Published:** 1902-12

**Authors:** 


### CORRESPONDENCE.

### The Maltine Company Prize Essay Awards.

*Editor Buffalo Medical Journal :*

SIR.—The two prizes, one of a $1,000 and another of $500, which we offered last January for the two best essays on Preventive Medicine have been awarded by the judges, Dr. Daniel Lewis, of New York; Dr. Charles A. L. Reed, of Cincinnati, and Dr. John E. Rhodes, of Chicago, who met for a final consultation at Buffalo. A facsimile of their letter of award is enclosed herewith. Two hundred and nine essays were submitted in competition, and although nearly every state in the Union was represented in the contest, both prizes were won by Philadelphia men.

The $1,000 prize was awarded to Dr. W. Wayne Babcock, 3302 North Broad Street, Philadelphia. His essay is entitled, The general principles of preventive medicine, and was submitted under the nom-de-plume, "Alexine."

The $500 prize was awarded to Dr. Lewis S. Somers, 3554 North Broad Street, Philadelphia. His essay is entitled, The Medical Inspection of Schools—a Problem in Preventive Medicine, and was submitted under the non-de-plume, "Broad."

The two successful essays will first be published in representative medical journals, and then in permanent form for gratuitous distribution to the profession at large.

The following tabulation will undoubtedly prove of interest to you and your readers. It shows how the various sections of the country were represented in the competition:

Alaska, 1; Arkansas, 1; California, 6; Colorado, 4; Connecticut, 5; District of Columbia, 3; Florida, 5; Georgia, 5; Illinois, 15; Indiana, 11; Iowa, 8; Kansas, 2; Kentucky, 3; Louisiana, 2; Maine, 4; Maryland, 2; Massachusetts, 12; Michigan, 7; Minnesota, 7; Mississippi, 1; Missouri, 5; Montana, 2; Nebraska, 2; New Hampshire, 1; New Jersey, 4; New York, 22; North Carolina, 1; Ohio, 11; Oregon, 1; Pennsylvania, 25; Rhode Island, 1; South Carolina, 2; Tennessee, 1; Texas, 2; Vermont, 1; Virginia, 1; Washington, 3; West Virginia, 2; Wisconsin, 10; Ontario, 2; New Brunswick, 1; Unidentified, 5; total, 209.

THE MALTINE CO.,
C. C. HEUMAN, Secretary.

NEW YORK CITY, BOROUGH OF BROOKLYN, October 31, 1902.

The following letter in facsimile accompanied the foregoing:

BUFFALO, October 18, 1902.

*To the Maltine Company, New York:*

GENTLEMEN.—Your committee selected to award the two prizes offered by your firm for essays on Preventive Medicine, or some subject connected therewith, begs leave to report that the large number offered in the competition (being two hundred and nine in all), and the general high grade of their excellence has made the matter of selection very difficult. After critical examination and mature deliberation, however, your committee has awarded:

The first prize to the essay entitled, The General Principles of Preventive Medicine, signed, "Alexine," and the second prize to the essay entitled, "The Medical Inspection of Schools —a Problem in Preventive Medicine, signed, "Broad."

In submitting this report the committee congratulates you upon the wide-spread interest which you have aroused in the very important subject of Preventive Medicine, and it congratulates the medical profession and the public upon the great good that will follow the publication of the valuable addition to literaure thus evoked by your enterprise.

Respectfully submitted,

DANIEL LEWIS,
CHARLES A. L. REED,
JOHN EDWIN RHODES,
Committee.

---

THE TREATMENT OF DYSMENNORRHEA.—This distressing and frequently obstinate complication of the menstrual function is often relieved by a prompt resort to Hayden's virburnum compound. In patients suffering from habitual dysmenorrhea it is well to commence by giving teaspoonful doses every night for a week previous to the usual time. Immediately on the appearance of the catamenia, a good plan is to give two teaspoonfuls every half hour in a wineglassful of hot water, sweetened, and continue to do so until relief is obtained, unless the stomach rebels, in which case the dose should be reduced until it is tolerated. In very severe cases and spasms, hot applications to the loins and inferior extremities are valuable aids as relaxants and in keeping up a healthy circulation. Frequently after taking viburnum compound, the patient will sleep soundly for several hours from the sudden cessation of pain; in such cases she should not be disturbed through any fear of over-sleeping, as the remedy does not contain any narcotic whatever, nor does it leave any disagreeable after-effect, and it may be given to a child, when necessary, without any special caution.

